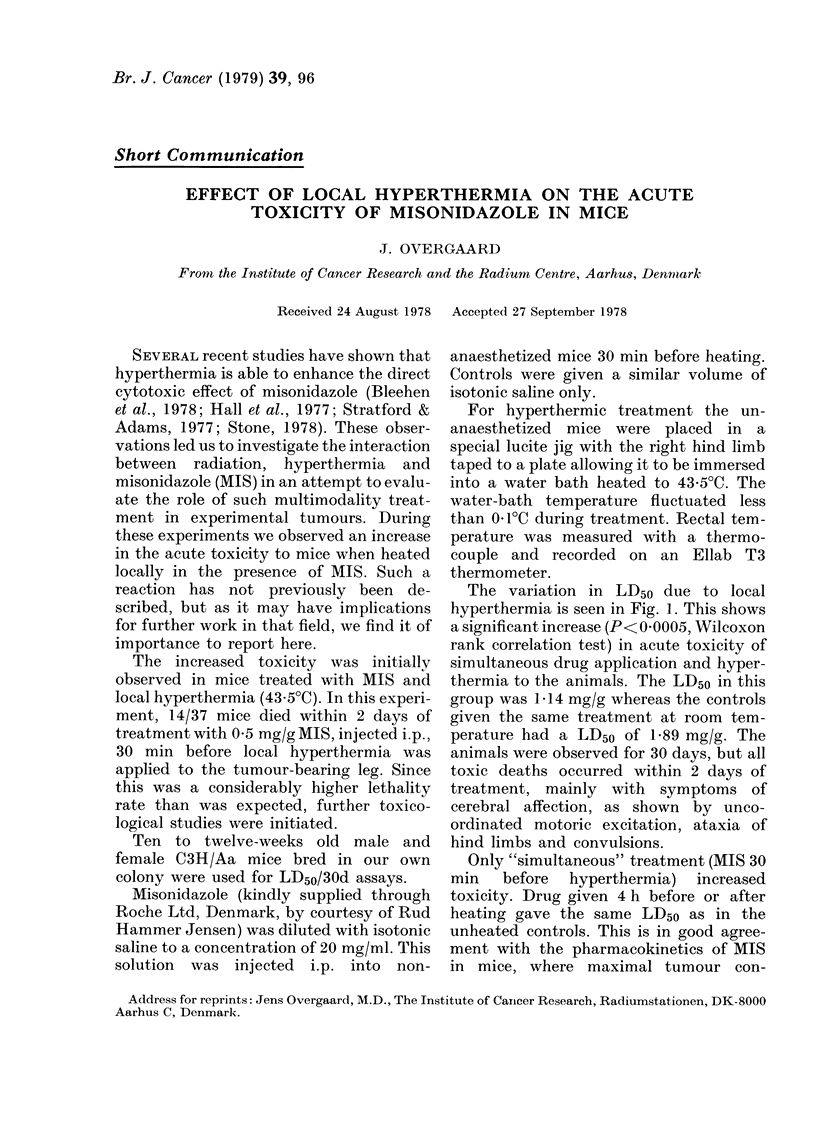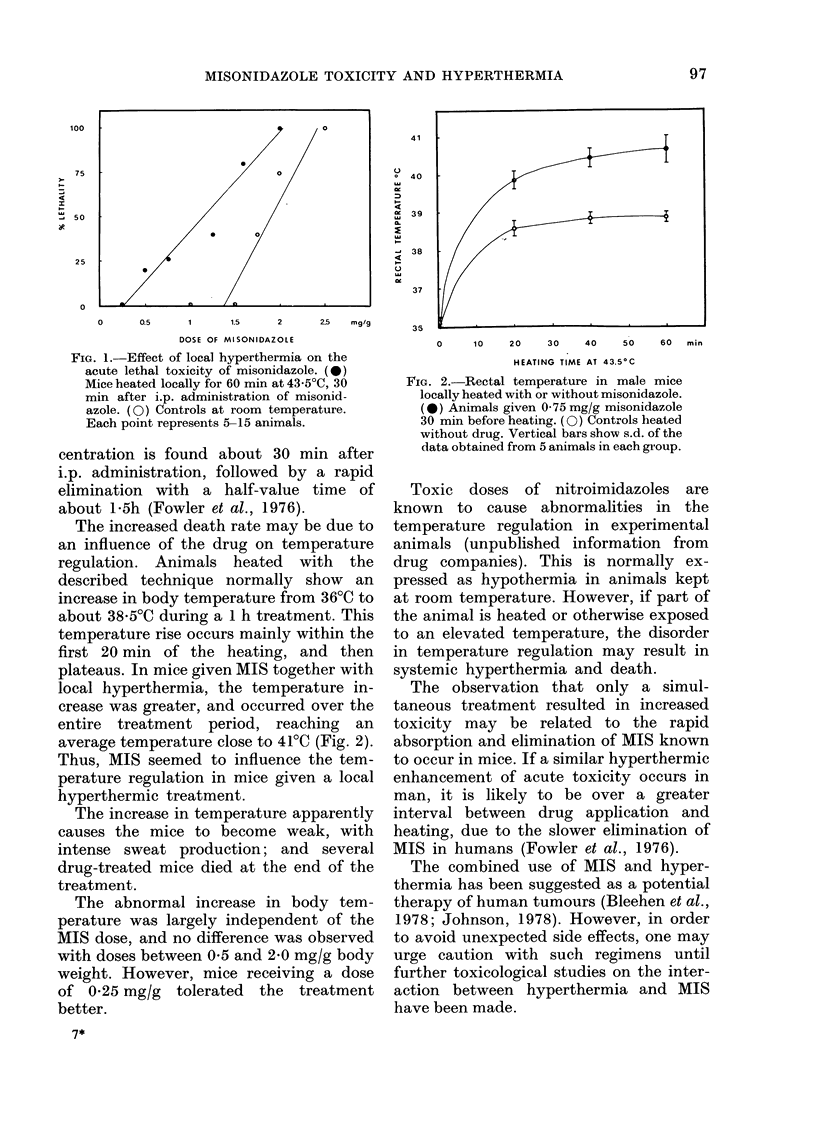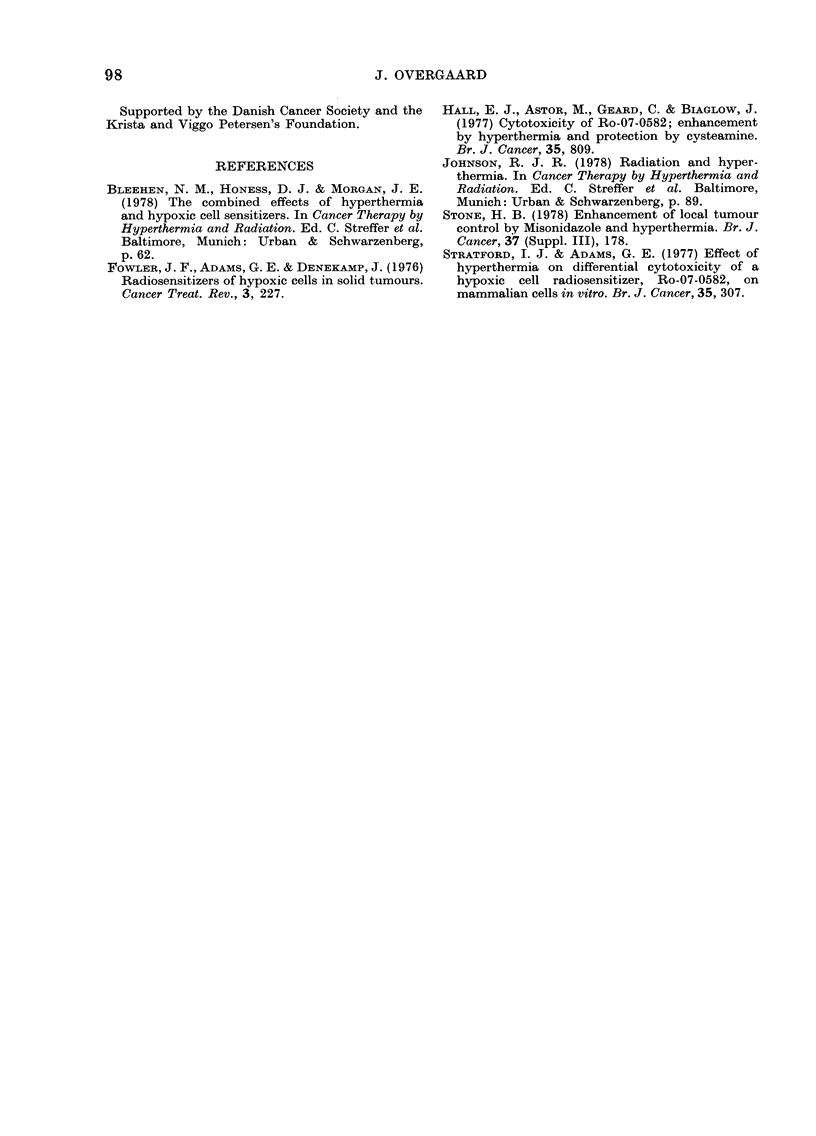# Effect of local hyperthermia on the acute toxicity of misonidazole in mice.

**DOI:** 10.1038/bjc.1979.12

**Published:** 1979-01

**Authors:** J. Overgaard


*Br. J. Cancer* (1979) **39**, 96

***Short Communication***

### EFFECT OF LOCAL HYPERTHERMIA ON THE ACUTE TOXICITY OF MISONIDAZOLE IN MICE

J. OVERGAARD

*From the Institute of Cancer Research and the Radium Centre, Aarhus, Denmark*

Received 24 August 1978 Accepted 27 September 1978

SEVERAL recent studies have shown that hyperthermia is able to enhance the direct cytotoxic effect of misonidazole (Bleehen *et al.*, 1978; Hall *et al.*, 1977; Stratford & Adams, 1977; Stone, 1978). These observations led us to investigate the interaction between radiation, hyperthermia and misonidazole (MIS) in an attempt to evaluate the role of such multimodality treatment in experimental tumours. During these experiments we observed an increase in the acute toxicity to mice when heated locally in the presence of MIS. Such a reaction has not previously been described, but as it may have implications for further work in that field, we find it of importance to report here.

The increased toxicity was initially observed in mice treated with MIS and local hyperthermia (43·5°C). In this experiment, 14/37 mice died within 2 days of treatment with 0·5 mg/g MIS, injected i.p., 30 min before local hyperthermia was applied to the tumour-bearing leg. Since this was a considerably higher lethality rate than was expected, further toxicological studies were initiated.

Ten to twelve-weeks old male and female C3H/Aa mice bred in our own colony were used for $LD_{50}$/30d assays.

Misonidazole (kindly supplied through Roche Ltd, Denmark, by courtesy of Rud Hammer Jensen) was diluted with isotonic saline to a concentration of 20 mg/ml. This solution was injected i.p. into non-anaesthetized mice 30 min before heating. Controls were given a similar volume of isotonic saline only.

For hyperthermic treatment the un-anaesthetized mice were placed in a special lucite jig with the right hind limb taped to a plate allowing it to be immersed into a water bath heated to 43·5°C. The water-bath temperature fluctuated less than 0·1°C during treatment. Rectal temperature was measured with a thermocouple and recorded on an Ellab T3 thermometer.

The variation in $LD_{50}$ due to local hyperthermia is seen in Fig. 1. This shows a significant increase ($P < 0{\cdot}0005$, Wilcoxon rank correlation test) in acute toxicity of simultaneous drug application and hyperthermia to the animals. The $LD_{50}$ in this group was 1·14 mg/g whereas the controls given the same treatment at room temperature had a $LD_{50}$ of 1·89 mg/g. The animals were observed for 30 days, but all toxic deaths occurred within 2 days of treatment, mainly with symptoms of cerebral affection, as shown by uncoordinated motoric excitation, ataxia of hind limbs and convulsions.

Only "simultaneous" treatment (MIS 30 min before hyperthermia) increased toxicity. Drug given 4 h before or after heating gave the same $LD_{50}$ as in the unheated controls. This is in good agreement with the pharmacokinetics of MIS in mice, where maximal tumour con-

Address for reprints: Jens Overgaard, M.D., The Institute of Cancer Research, Radiumstationen, DK-8000 Aarhus C, Denmark.

FIG. 1.—Effect of local hyperthermia on the acute lethal toxicity of misonidazole. (●) Mice heated locally for 60 min at 43·5°C, 30 min after i.p. administration of misonidazole. (○) Controls at room temperature. Each point represents 5–15 animals.

FIG. 2.—Rectal temperature in male mice locally heated with or without misonidazole. (●) Animals given 0·75 mg/g misonidazole 30 min before heating. (○) Controls heated without drug. Vertical bars show s.d. of the data obtained from 5 animals in each group.

centration is found about 30 min after i.p. administration, followed by a rapid elimination with a half-value time of about 1·5h (Fowler *et al.*, 1976).

The increased death rate may be due to an influence of the drug on temperature regulation. Animals heated with the described technique normally show an increase in body temperature from 36°C to about 38·5°C during a 1 h treatment. This temperature rise occurs mainly within the first 20 min of the heating, and then plateaus. In mice given MIS together with local hyperthermia, the temperature increase was greater, and occurred over the entire treatment period, reaching an average temperature close to 41°C (Fig. 2). Thus, MIS seemed to influence the temperature regulation in mice given a local hyperthermic treatment.

The increase in temperature apparently causes the mice to become weak, with intense sweat production; and several drug-treated mice died at the end of the treatment.

The abnormal increase in body temperature was largely independent of the MIS dose, and no difference was observed with doses between 0·5 and 2·0 mg/g body weight. However, mice receiving a dose of 0·25 mg/g tolerated the treatment better.

Toxic doses of nitroimidazoles are known to cause abnormalities in the temperature regulation in experimental animals (unpublished information from drug companies). This is normally expressed as hypothermia in animals kept at room temperature. However, if part of the animal is heated or otherwise exposed to an elevated temperature, the disorder in temperature regulation may result in systemic hyperthermia and death.

The observation that only a simultaneous treatment resulted in increased toxicity may be related to the rapid absorption and elimination of MIS known to occur in mice. If a similar hyperthermic enhancement of acute toxicity occurs in man, it is likely to be over a greater interval between drug application and heating, due to the slower elimination of MIS in humans (Fowler *et al.*, 1976).

The combined use of MIS and hyperthermia has been suggested as a potential therapy of human tumours (Bleehen *et al.*, 1978; Johnson, 1978). However, in order to avoid unexpected side effects, one may urge caution with such regimens until further toxicological studies on the interaction between hyperthermia and MIS have been made.

Supported by the Danish Cancer Society and the Krista and Viggo Petersen's Foundation.

### REFERENCES

BLEEHEN, N. M., HONESS, D. J. & MORGAN, J. E. (1978) The combined effects of hyperthermia and hypoxic cell sensitizers. In *Cancer Therapy by Hyperthermia and Radiation*. Ed. C. Streffer *et al.* Baltimore, Munich: Urban & Schwarzenberg, p. 62.

FOWLER, J. F., ADAMS, G. E. & DENEKAMP, J. (1976) Radiosensitizers of hypoxic cells in solid tumours. *Cancer Treat. Rev.*, **3**, 227.

HALL, E. J., ASTOR, M., GEARD, C. & BIAGLOW, J. (1977) Cytotoxicity of Ro-07-0582; enhancement by hyperthermia and protection by cysteamine. *Br. J. Cancer*, **35**, 809.

JOHNSON, R. J. R. (1978) Radiation and hyperthermia. In *Cancer Therapy by Hyperthermia and Radiation*. Ed. C. Streffer *et al.* Baltimore, Munich: Urban & Schwarzenberg, p. 89.

STONE, H. B. (1978) Enhancement of local tumour control by Misonidazole and hyperthermia. *Br. J. Cancer*, **37** (Suppl. III), 178.

STRATFORD, I. J. & ADAMS, G. E. (1977) Effect of hyperthermia on differential cytotoxicity of a hypoxic cell radiosensitizer, Ro-07-0582, on mammalian cells *in vitro*. *Br. J. Cancer*, **35**, 307.